# Identification and survival studies of *Mycobacterium tuberculosis* within Laboratory-Fermented bovine milk

**DOI:** 10.1186/1756-0500-7-175

**Published:** 2014-03-26

**Authors:** Solomon H Mariam

**Affiliations:** 1 Armauer Hansen Research Institute (AHRI), P.O. Box 1005, Addis Ababa, Ethiopia; 2 Aklilu Lemma Institute of Pathobiology, Addis Ababa University, Addis Ababa, Ethiopia

**Keywords:** Lactic acid bacteria, *Mycobacterium tuberculosis*, Fermented milk, Inhibition

## Abstract

**Background:**

*Mycobacterium tuberculosis* and *Mycobacterium bovis* are the classic agents causing tuberculosis (TB) in humans and animals respectively. Transmission of tuberculous bacteria to humans usually occurs by inhalation of aerosols containing droplets of tubercle bacilli or via consumption of contaminated foods and drinks, primarily milk. The practice of milk pooling, including from cows with TB of the udder, further exacerbates the situation by rendering the whole milk supply infective. The simultaneous presence of indigenous lactic acid bacteria (LAB) in Mycobacterium-contaminated milk is believed to confer protective effect when the milk is adequately fermented. This study assessed the effect of LAB on the viability of mycobacteria in inherently contaminated pool of raw milk during fermentation as a function of time.

**Findings:**

Growth was obtained in the pooled raw milk culture, and identified to be *M. tuberculosis*. This *M. tuberculosis* growth was undetectable in the milk culture by day 7 as assessed by plating serial dilutions of the milk culture for up to 14 days.

**Conclusions:**

Some LAB species appear to show inhibitory effect on tubercle bacilli. If proven by more rigorous, controlled experimental results regarding such effect, selected LAB (with proven safety and efficacy) may have potential applications as anti-mycobacterial agents.

## Findings

### Background

While *Mycobacterium tuberculosis* and *Mycobacterium bovis* are the classic agents causing human and bovine tuberculosis (TB) respectively, there have been instances of the isolation of *M. tuberculosis* as well as *M. bovis* from bovine tissues and milk [1-3]. There is widespread infection with bovine tuberculosis in Ethiopian cattle [2,4,5]. These two tubercle bacilli can be transmitted between humans and animals in two-way fashion directly (i.e., via aerosol inhalation) or indirectly from animals to humans (i.e., by consumption of raw contaminated dairy products and meat) [6,7]. Milking cows with TB of the udder may also act as amplifiers of TB by rendering the whole milk supply infective in places where milk pooling is practiced. Consumption of such foods is likely to cause extrapulmonary TB (EPTB).

Neither the diagnosis of EPTB nor the distinction whether TB is caused by *M. tuberculosis* or *M. bovis* is possible by chest radiography, sputum smear microscopy and sputum culture. Misdiagnosis of *M. bovis* infection for *M. tuberculosis* or vice versa can have important consequences, including the wrong treatment with pyrazinamide in the case of the former. Reports of the World Health Organization continue to show that the notification rate of EPTB in Ethiopia is very high (35% of new cases) [8].

Lactic acid bacteria (LAB) are indigenously found in various habitats including milk, meats and the mucosal surfaces of animals [9,10]. LAB have been used for centuries for preservation and bioconversion of various foods and drinks (e.g., production of yogurt, wine, cheese, beverages, sauerkraut, etc). They also have important industrial applications in the making of fermented foods and drinks. Many of the LAB species are nonpathogenic and safe, at least in healthy and immune-competent people, and some have been designated as probiotics. These species have several claimed benefits, one of which is their potential for use as anti-infective agents [11-13].

Several reports indicate the potential of selected LAB species, among their other beneficial effects, in inhibiting pathogenic bacteria such as *Helicobacter pylori*[14-18], *Listeria monocytogenes*[19,20], *Salmonella enterica*[21] and *Clostridium* species [20,22,23] by various mechanisms including inhibition of bacterial adhesion, enhancement of host immunity and production of antimicrobial substances.

In Ethiopian country households, fresh milk is traditionally allowed to ferment at room temperature. Fermented milk can be stored and consumed for up to 20 days. Moreover, raw fresh and fermented milk is consumed widely [24]. Consumption of raw, contaminated milk is a risk factor for TB [25,26].

This study aimed both to identify and evaluate the survival of mycobacteria in milk fermented in the laboratory under conditions simulating traditional household milk fermentation. The study involved milk culture in which the fate of inherently-present mycobacteria could be assessed by periodic plating on defined, selective solid medium.

## Materials and Methods

### The study site and milk collection

The site from which the milk sample used in this experiment was obtained is located 30 kms west of the Ethiopian capital, Addis Ababa. It is a State Dairy Farm with herd size of 200 cows. The farm serves as one of the major suppliers of milk to the city, to which milk from all lactating cows is pooled into large containers and sent. A one-liter pooled milk sample was obtained from 30 tuberculin skin-test positive cows from the farm. The milk was collected hygienically into sterile containers and transported to AHRI within a cold chain. The milk was maintained within dark bottle in a cardboard box at room temperature for the duration of the experiment.

### Antibiotics for selective inhibition of LAB

To inhibit the growth of LAB during enumeration of mycobacteria, an antibiotic cocktail was incorporated into Middlebrook 7H10 agar medium as described previously [27].

### Enumeration of mycobacteria

Enumeration of *mycobacteria* at time intervals 0, 3, 7 and 14 days was performed as described previously [27].

### Identification of mycobacteria from pooled milk

Tests used for identification of unknown bacteria from the milk culture included acid-fast staining, catalase test and PCR. For PCR, a 2X master mix from Qiagen was used. The PCR reactions were multiplex PCR using three sets of primers: Mycgen-F, Mycgen-R, Mycav-R; RD4F, RD4R, RD4intF; and RD10F, RD10R, RD10intR [28]. Mycgen-F and Mycgen-R are specific for the genus *Mycobacterium* and give a product size of 1030 bp. Mycgen-F together with Mycav-R is specific for *Mycobacterium avium* with a product size of 180 bp. The cycling conditions were an initial denaturation at 95°C for 15 min followed by 35 cycles of denaturation at 95°C for 1 min, annealing at 61°C for 30 s and extension at 72°C for 2 min with final extension at 72°C for 7 min. With the RD4 multiplex, a product size of 446 bp is expected if it is *M. bovis* and 335 bp if it is *M. tuberculosis*. With RD10, a product size of 202 bp is expected if it is *M. bovis* and 308 bp if it is *M. tuberculosis*. The cycling conditions for both RD4 and RD10 primers were initial denaturation at 95°C for 15 min followed by 35 cycles of denaturation at 95°C for 1 min, annealing at 55°C for 1 min and extension at 72°C for 1 min with final extension at 72°C for 10 min.

## Results and Discussion

### Bacterial growth from pooled milk culture and pH of fermented milk

From the pooled milk culture, growth was obtained on 7H10 plates. It was later found to be acid-fast-positive. Tests conducted to assess the catalase activity of this bacterial growth yielded catalase-positive results. There were 4.7 ± 4.4 and 3.9 ± 3.7 Log CFU mL^-1^ on days zero (start of the milk culture) and 3 respectively. This mycobacterial growth was subject to elimination by indigenous LAB after 7 days.

The pH of milk was measured by saving 50 mL of the milk sample for this purpose. The pH changed from ~ 7.00 on day zero to 4.59 on day 8, with the day zero value being dependent on how fast after milk collection the pH was measured.

### Identification of bacterial growth from pooled milk culture by PCR

**Figure 1:**
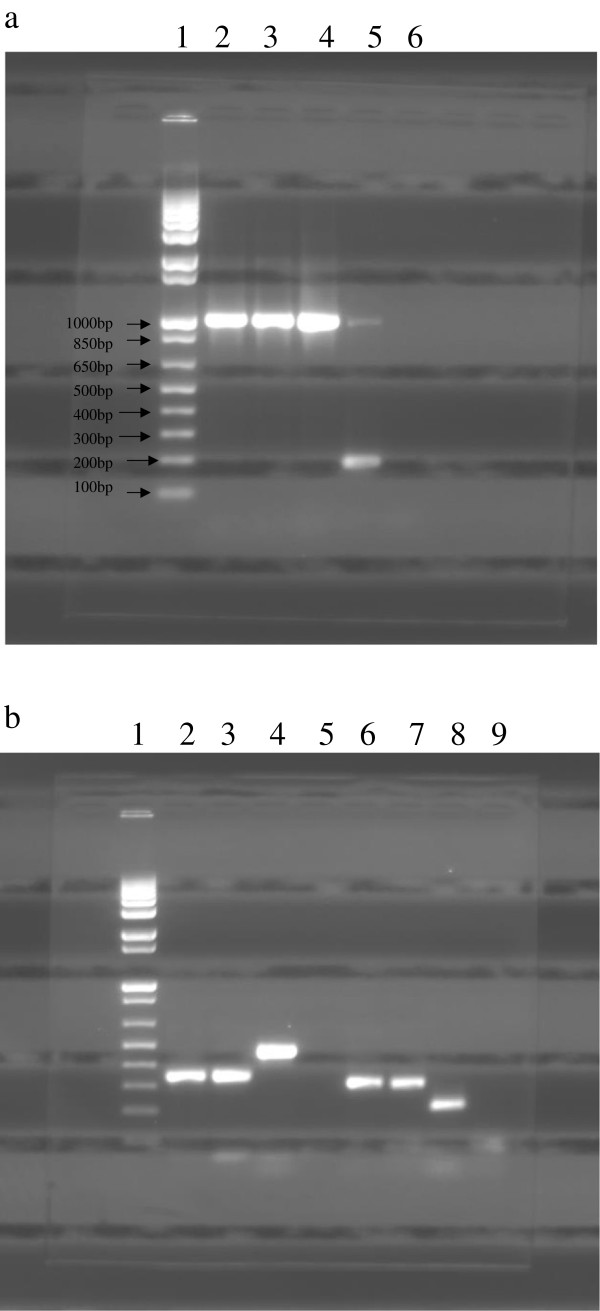
**Detection of milk isolate as *M. tuberculosis *by Genus- and species-specific PCR. (a)** Multiplex PCR with primers Mycgen-F, Mycgen-R and Mycav-R. Lane 1: 1 Kb Plus DNA ladder, lane 2: milk isolate, lanes 3-5: positive controls with *M. tuberculosis*, *M. bovis* and *M. avium* DNA respectively, lane 6: negative control. **(b)** Multiplex PCR with RD4 (lanes 2-5) and RD10 (lanes 6-9) primers. Lane 1: 1 Kb Plus DNA ladder, lane 2: milk isolate, lanes 3 and 4: positive controls with *M. tuberculosis* and *M. bovis* DNA respectively, lane 5: negative control. Lane 6: milk isolate, lanes 7 and 8: positive controls with *M. tuberculosis* and *M. bovis* DNA respectively, lane 9: negative control. Molecular weight ladder in b same as in a.

The isolate from the milk culture, following molecular characterization by PCR, gave a product size of 1030 bp with the *Mycobacterium* genus-specific primers (Figure 1a). The species-level identification with the RD4 deletion primers gave a product size of 335 bp while with the RD10 deletion primers it gave a product size of 308 bp, both typical of *M. tuberculosis* DNA amplification products (Figure 1b), indicating the presence of *M. tuberculosis* in the pooled milk sample. The PCR results with the genus-specific and deletion primers also confirmed that the milk isolate was neither *Mycobacterium avium* nor *Mycobacterium africanum*.

#### *Impact of milk mycobacteria on human health*

*M. tuberculosis* was isolated from pooled milk. Although cow’s milk is most common, milk from other animals (goats, camels) is also consumed by the population existing in the lowlands of Ethiopia, where livestock breeding is the major livelihood. Other studies [2,4,29] showed that there is widespread occurrence of mycobacterial infection in livestock in many areas of Ethiopia. Milk from these sources is most commonly consumed raw. Practical problems include the impracticability of boiling milk before consumption in most household conditions, the absence of pasteurization, and the inefficacy of pasteurization to eliminate all pathogens, assuming it was applied.

The presence of *Mycobacteria* in bovine milk is risky for two major reasons: (i) it becomes enough to contaminate with bacterial pathogens when and where milk pooling is practiced, even if the milk from only one of the cows was contaminated while the others were free of contamination; (ii) in the case of milk fermentation, adding fresh milk to already-fermenting milk (which is the practice in most Ethiopian farmers’ households) [24] becomes reason for the continued presence of such bacteria in the fermented milk. Thus, for the fermentation process to be adequate to eliminate the mycobacteria, the milk should be kept fermenting at room temperature for the duration of fermentation without addition of fresh milk in between.

It can be seen that there is a significant decrease in the number of *M. tuberculosis* CFU after seven days. However, this is an issue of not significant reductions in CFU. This is “zero tolerance” and the pathogen must be eliminated completely for the milk to be safe for consumption. There is no threshold level below which there is an acceptable level of contamination. Significant reductions in CFU, however high they are, are not guarantee for absence of acquisition of infection. Although the infectious dose of *M. tuberculosis* by the gastrointestinal route is much higher than that by the respiratory route (ID_50_ < 10 bacilli) [30,31], the number of *M. tuberculosis* detected in the milk sample in this work during the first three days of the fermentation (≥ 4 Logs) is high enough to meet that high dose, assuming a person frequently consumes such milk.

#### *Limitations of the Study and Remarks on Further Work*

Although the elimination of *M. tuberculosis* was observed in two independent experiments in this work, it is not yet warranted, based on the results of this study alone, to make the recommendation that *M. tuberculosis*-contaminated milk will be safe for consumption if fermented for a defined period of time. Before such a recommendation can be made, large-scale controlled studies on naturally-contaminated milk samples would need to be carried out to verify the elimination of *M. tuberculosis*. These studies will have to take into account all possible confounding factors including: (i) the effect of the practice of adding fresh contaminated milk to already fermenting milk, (ii) effect of the volume of milk and how this might influence the rate of elimination, or survival, of *M. tuberculosis*, (iii) the rate of elimination or survival of *M. tuberculosis* in relation to the useful shelf-life of the fermented milk, (iii) the relative numbers of contaminating *M. tuberculosis* and numbers and types (i.e., identification to at least genus and species level) of the resident LAB and how this affects the rate of elimination, or survival, of *M. tuberculosis*. Identifying possible sources of contamination will also be important, as well as conditions for pre- and post-processing handling of milk, because sometimes contamination can come from external sources (e.g., milking equipment or utensils, the teats of the animals, hands). The studies will also need to take into account the influence of prevailing temperatures in different geographical localities on the rate of elimination, or survival, of *M. tuberculosis*.

## Conclusions

The elimination of *M. tuberculosis* in raw, fermented milk appears to be attributable to the resident LAB microflora. Among the mechanisms by which LAB inhibit pathogens in foods are production of lactic acid and bacteriocins and consequently, LAB are being used in the food industry as starter cultures, for enhancement of food safety and extension of shelf-life [19,32,33]. The elimination of *M. tuberculosis*, if proven by more rigorous experiments, may have two implications: first, if *M. tuberculosis* is completely eliminated, it means adequately fermented milk, free of *M. tuberculosis* may be safe for consumption; second, and pending proof of their safety and efficacy with respect to their multifaceted effects, the presence of LAB in fermented milk may be useful as they may also have probiotic effects.

## Abbreviations

TB: Tuberculosis; LAB: Lactic acid bacteria; EPTB: Extrapulmonary tuberculosis; AHRI: Armauer Hansen Research Institute; PCR: Polymerase chain reaction; CFU: Colony-forming units.

## Competing interests

The author declared that he has no competing interests.
